# 

**Published:** 1802-01

**Authors:** 


### THE MEDICAL AND PHYSICAL JOURNAL,

CONTAINING

THE EARLIEST INFORMATION

ON SUBJECTS OF

MEDICINE, | SURGERY,

PHARMACY, | CHEMISTRY,

AND

NATURAL HISTORY;

AND A

CRITICAL ANALYSIS OF ALL NEW WORKS

*In those DEPARTMENTS of LITERATURE.*

CONDUCTED BY

T. BRADLEY, M.D.

Member of the Royal College of Phyſicians, London; Phyſician to the Weſtminſter Hoſpital, and to the Aſylum for Female Orphans; Lecturer on the Theory and Practice of Medicine, &c.

BY

R. BATTY, M.D.

Licentiate in Midwifery, of the Royal College of Phyſicians, London; Phyſician to the Britiſh Lying-In-Hoſpital, Brownlow Street; and to the Infant Aſylum; Fellow of the Linnean Society; and Lecturer on Midwifery.

AND BY

A. A. NOEHDEN, M.D.

Vice-Secretary of the Phyſical Society of Gottingen; Correſponding Member of the Linnean Society, London, and of the Medical Society at Paris.

VOL. VII.

FROM JANUARY TO JUNE, 1802.

Surgeon Gen'l's Office LIBRARY. 5713 Washington, D.C.

*LONDON:*

Printed for R. PHILLIPS, No. 71, ST. PAUL'S CHURCH YARD; and sold by all Booksellers in the United Kingdoms.

*Price* 15 *s.* 6 *d. in Boards.*

[W. THORNE, Printer, Red Lion Court, Fleet Street.]

[ Entered at Stationers Hall. ]

W
1
ME 182

Medical and Physical Memoirs, containing among other subjects, a particular Enquiry into the Nature of the Pestilential Empidema of the United States, by CHARLES CALDWELL, M. D. 8vo. 8s. boards Wynne and Scholey.

Experiments and Observations on the Mineral Waters of Hampstead and Kilburn. By JOHN BLISS, Surgeon, 2s. Phillips.

New Inventions and Directions for Ruptured Persons, by W. H. T. Esq. with a Recommendatory Letter from WILLIAM BLAIR, A. M. Surgeon. 2d Edition with Additions, 2s. Hurst.

Observations on the Opinion of Dr. Langslow, that Extravasation is the general cause of Apoplexy. By WILLIAM CROWFOOT. Robinsons.

Medicinæ Praxeos Compendium Symptomata, Causas, Diagnosin, Prognosin, et medendi rationem exhibens. Autore, E. G. CLARKE, M. D. 5s. 6d. Edit. 2da. Ogle.

Account of the Plan for the Improvement and Extention of the Infirmary at Newcastle. Walker, Newcastle.

Historical Surgery, or the Progress of the Science of Medicine on Inflammation, Mortification, and Gun-shot Wounds, by JOHN HUNT. 4to. 1l. 1s. boards. Rivingtons

Practical Observations on the Gonorrhœa Virulenta, and a New Mode of Treating that Disease. By ROBERT BARKER. 2s. 6d. Rivingtons.

A Treatise on the new discoverd Dropsy of the Membranes of the Brain, and Watery Head of Children, proving that it may be cured. With Objections to Vomits, &c. To which are added, Observations on Errors in Nursing, &c. &c. By WILLIAM ROWLEY, M. D. 2s. Murray and Highley.

The Anatomist's Vade Mecum, the fourth Edition, revised and enlarged. By ROBERT HOOPER, M. D. 12mo. 7s. 6d. boards. Murray and Highley.

---

### To CORRESPONDENTS.

*We are particularly indebted to Dr. Girdlestone for his polite attention to our* Quere *respecting the prevalence of* Typhus *in autumn, (see p.* 54). *It appears to us, that a more accurate knowledge of the causes, and therefore of the means of preventing contagious and epidemical diseases, may be promoted by correct answers to similar questions from every part of the world. We have, at present, limited our inquiries to Idiopathic continued fevers, which we have attempted to distinguish under the Diseases admitted into the Westminster Hospital, (see p.* 16) *but mean hereafter to extend them to Scarlatina, Pertussis, Croup, Influenza, &c. We shall be particularly obliged to those Correspondents who add the state of the weather, soil, diet, &c. accompanying each Epidemic.*

*We are again obliged to apologize for postponing the account of books; but it shall not be deferred beyond the next Number.*

Communications *have been received from Dr. Brown, Mess. Ward, Howard, Wilkinson, and Observator.*

